# Jahresbericht über die Fortschritte auf dem Gebiete der Chirurgie

**Published:** 1898-03

**Authors:** 


**Jahresbericht über die Fortschritte auf dem Gebiete der Chirurgie.** II. Jahrgang, 1896. Redigirt und herausgegeben von Prof. Dr. Hildebrand. Wiesbaden: J. F. Bergmann. 1897.

This is a work of some thirteen hundred pages on the progress of surgery in the year 1896. To those who are accustomed to such modest compendiums as Braithwaite, or Cassell's or Wright's Annual, which profess to contain accounts of the advance of medical science in all its branches within the space of a few hundred pages, this volume may seem at first sight too bulky and immense to be of use in their work. But on looking into it, it is soon seen how exceedingly useful it is; for we find abstracts (in German) of the best papers on surgery, published in the year 1896, in Russia, Germany, Italy, France, Spain, and English-speaking countries. These abstracts are classified under their respective heads. In addition there are notices of all books on surgery brought out during the year. The result naturally is that the volume is a most valuable work of reference. Suppose, for instance, that we wish to find what work has recently been done in the domain of intracranial complications of middle-ear disease, there are to be found here abstracts of sixty-four papers published in the year.

There is a complete double index, one of names and the other of diseases and organs. The volume is a splendid example of the thoroughness of German scientific work, and of its eminently practical and useful results.

